# Ketamine augmentation of electroconvulsive therapy to improve neuropsychological and clinical outcomes in depression (Ketamine-ECT): a multicentre, double-blind, randomised, parallel-group, superiority trial

**DOI:** 10.1016/S2215-0366(17)30077-9

**Published:** 2017-05

**Authors:** Ian M Anderson, Andrew Blamire, Tim Branton, Ross Clark, Darragh Downey, Graham Dunn, Andrew Easton, Rebecca Elliott, Clare Elwell, Katherine Hayden, Fiona Holland, Salman Karim, Colleen Loo, Jo Lowe, Rajesh Nair, Timothy Oakley, Antony Prakash, Parveen K Sharma, Stephen R Williams, R Hamish McAllister-Williams, Ketamine-ECT Study team, Claire Blakeley, Katherine Crosby, Aisha Perkis, Graham Spencer, Liam Trevithick, Amanda Watson, Francesca Williams, Audrey Williamson

**Affiliations:** a Neuroscience and Psychiatry Unit, University of Manchester, Manchester Academic Health Science Centre, Manchester, UK; b Institute of Cellular Medicine, Newcastle University, Newcastle upon Tyne, UK; c Leeds and York Partnership NHS Foundation Trust, Leeds, UK; d Central Manchester University Hospitals NHS Foundation Trust, Manchester, UK; e Centre for Biostatistics, University of Manchester, Manchester Academic Health Science Centre, Manchester, UK; f Nuffield Health, Leeds, UK; g Biomedical Optics Research Laboratory, University College London, London, UK; h Pennine Care NHS Foundation Trust, Stockport, UK; i Lancashire Care NHS Foundation Trust & University of Manchester, Preston, UK; j School of Psychiatry, University of New South Wales, Black Dog Institute & St George Hospital, Sydney, NSW, Australia; k Tees, Esk and Wear Valley NHS Foundation Trust, Darlington, UK; l Northumberland Tyne and Wear NHS Foundation Trust, Newcastle upon Tyne, UK; m Derbyshire Healthcare NHS Foundation Trust, Derby, UK; n Manchester Mental Health and Social Care Trust, Manchester, UK; o Centre for Imaging Science, University of Manchester, Manchester Academic Health Science Centre, Manchester, UK; p Institute of Neuroscience, Newcastle University, Newcastle upon Tyne, UK

## Abstract

**Background:**

The use of electroconvulsive therapy (ECT) is limited by concerns about its cognitive adverse effects. Preliminary evidence suggests that administering the glutamate antagonist ketamine with ECT might alleviate cognitive adverse effects and accelerate symptomatic improvement; we tested this in a randomised trial of low-dose ketamine.

**Methods:**

In this multicentre, randomised, parallel-group study in 11 ECT suites serving inpatient and outpatient care settings in seven National Health Service trusts in the North of England, we recruited severely depressed patients, who were diagnosed as having unipolar or bipolar depressive episodes defined as moderate or severe by DSM-IV criteria, aged at least 18 years, and were able and willing to provide written consent to participate in the study. Patients were randomly assigned (1:1) to ketamine (0·5 mg/kg intravenous bolus) or saline adjunctive to the anaesthetic for the duration of their ECT course. Patients and assessment and ECT treatment teams were masked to treatment allocation, although anaesthetists administering the study medication were not. We analysed the primary outcome, Hopkins Verbal Learning Test-Revised delayed verbal recall (HVLT-R-DR) after four ECT treatments, using a Gaussian repeated measures model in all patients receiving the first ECT treatment. In the same population, safety was assessed by adverse effect monitoring. This trial was registered with International Standard Randomised Controlled Trial Number, number ISRCTN14689382.

**Findings:**

Between early December, 2012, and mid-June, 2015, 628 patients were screened for eligibility, of whom 79 were randomly assigned to treatment (40 in the ketamine group *vs* 39 in the saline group). Ketamine (mean 5·17, SD 2·92), when compared with saline (5·54, 3·42), had no benefit on the primary outcome (HVLT-R-DR; difference in means −0·43 [95% CI −1·73 to 0·87]). 15 (45%) of 33 ketamine-treated patients compared with 10 (27%) of 37 patients receiving saline experienced at least one adverse event which included two (6%) of 33 patients who had ketamine-attributable transient psychological effects. Psychiatric adverse events were the most common in both groups (six [27%] of 22 adverse events in the ketamine group *vs* seven [54%] of 13 in the saline group).

**Interpretation:**

No evidence of benefit for ketamine was found although the sample size used was small; however, the results excluded greater than a small to moderate benefit with 95% confidence. The results do not support the use of adjunctive low-dose ketamine in routine ECT treatment.

**Funding:**

National Institute for Health Research (NIHR) Efficacy and Mechanism Evaluation (EME) programme, an MRC and NIHR partnership.

## Introduction

The naturalistic STAR*D study^1^ found that about a third of patients with depression did not remit even after four sequential pharmacological treatments.^1^ The National Institute for Health and Care Excellence (NICE) recommends electroconvulsive therapy (ECT) as a treatment option for patients with moderate or severe depression if they have not responded to multiple drug and psychological treatments.^2^ ECT has greater acute effectiveness than pharmacotherapy with a large effect size against sham treatment of −0·8,^3^ and acute remission rates of just under 50% in patients not responding to previous drug treatments.^4^ Scottish ECT audit data for 2014^5^ reported that about 75% of patients with depression, who were most resistant to previous treatment, had a good clinical response to ECT.^5^

The number of patients receiving ECT has fallen substantially from around 20 000 a year in England and Wales in the 1980s^6^ to under 5000 by 2006,^7^ with Scottish audit data also showing a continuing gradual decline.^5^ A major factor in this decline appears to be concern about a poor risk-benefit balance of ECT because of adverse cognitive side-effects.^8^ A meta-analysis of 84 studies^9^ found that ECT has moderate to large adverse effects on cognition when measured up to 3 days after the final treatment, especially on anterograde memory, executive function, and cognitive processing speed.^9^ Most deficits reverse by 1–2 weeks after the final ECT treatment,^9^ but it is unknown whether persisting adverse effects from ECT are masked by positive effects from clinical improvement. One uncertainty is whether retrograde amnesia or loss of autobiographical memories following ECT is persistent,^10^, ^11^ with a systematic review of seven studies^8^ of patient self-reports finding that persistent loss of memories after ECT varied from 29% to 55% in individual studies.

**Research in context**

**Evidence before this study**

The Technology Appraisal of electroconvulsive therapy (ECT) by the National Institute for Health and Care Excellence (NICE) in 2003 and the updated NICE Clinical Guideline for the treatment of Depression (2009) identified ECT as highly effective in the acute treatment of depression but that its use was limited by cognitive adverse effects, particularly memory impairment. Theoretical, animal, and preliminary, non-randomised human study evidence suggested that the glutamate antagonist ketamine might alleviate the adverse cognitive effects of ECT. A series of small randomised trials had identified ketamine as having a rapid antidepressant effect and it had been hypothesised that it might enhance the therapeutic effect of ECT. We searched PubMed with the terms “ketamine” and “electroconvulsive therapy” or “ECT” for articles published in English up until Nov 30, 2016. We found 12 randomised trials of varying quality in which ketamine had been used as an alternative or adjunctive anaesthetic agent compared with a barbiturate anaesthetic or propofol. Three small randomised trials reported time to reorientation after ECT and found conflicting results. A moderately large study reported a benefit for lower dose of ketamine than used in the reported study in addition to propofol on a range of aspects of memory. Two smaller randomised trials used several cognitive tests, both finding no effect of ketamine as an augmenting agent, but in one of the trials less impairment was found comparing ketamine alone with propofol on selected tests of executive function without effect on memory. One study reported a better outcome on the Mini Mental State Examination on ketamine compared with thiopental but the result was not statistically significant. Several studies suggest that ketamine might lead to longer seizures with ECT, and increase agitation or confusion on awakening, especially when given at anaesthetic doses.

**Added value of this study**

Our study was the first multicentre randomised trial of adjunctive sub-anaesthetic ketamine on the effect of bilateral brief-pulse ECT as administered in the UK on verbal anterograde and autobiographical memory. Its strengths are the generalisability to standard UK practice with a robust design and analysis. The study adds to evidence from three other studies with varying methods that investigated the effect of ketamine on cognitive function during ECT assessed using several neuropsychological tests. We did not find any evidence for an advantage for ketamine treatment on any cognitive or efficacy outcomes, with a numerical advantage for the saline-treated group on delayed verbal memory (the primary outcome) and early and final reduction in depression scores. In spite of lower than planned recruitment, we were able to exclude more than a small-to-moderate effect size benefit for ketamine on the key outcome measures. A minority of patients experienced ketamine-related psychological effects on awakening from ECT but no serious safety or tolerability problems were found.

**Implications of all the available evidence**

The literature, involving studies with varying methods, is inconsistent with regard to whether, and in what specific domains, ketamine has benefit when combined with ECT for the treatment of depression. The balance of evidence for ketamine used as an adjunctive treatment with ECT at the most common dose of 0·5 mg/kg is that it does not improve the cognitive effect of, or the therapeutic response to, ECT to a clinically important degree. However, although the evidence does not support giving low-dose ketamine as part of routine ECT practice as administered in the UK, it does not rule out its possible benefit on cognition in specific patient populations or with different ketamine or ECT regimens.

Cognitive impairment and effectiveness of treatment are both related to the stimulus dose, especially for right unilateral ECT,^2^, ^3^ and there is a clinical trade-off between the two effects; a meta-analysis^12^ found that high-dose right unilateral ECT was as effective as moderate-dose bitemporal ECT, with a similar cognitive effect profile for anterograde memory and verbal fluency; however, right unilateral ECT might have benefits for retrograde autobiographical memory and reorientation time after seizure. Ultra-brief pulse right unilateral ECT (pulse width 0·3 ms) was associated with less cognitive impairment but was also less effective than brief pulse right unilateral ECT (pulse width 1·0–1·5 ms).^13^ At present, the best strategy to maintain the efficacy of ECT while minimising cognitive impairment has not been established clinically.

The neurotransmitter glutamate has a central role in cognition, especially in learning and memory, through its effects on synaptic plasticity and the signalling pathway involved in long-term potentiation in the hippocampus.^14^ Memory impairment after ECT could be a consequence of indiscriminate activation or saturation of glutamate receptors during the treatment, disrupting hippocampal plasticity involved in memory.^15^ Ketamine, a dissociative anaesthetic, analgesic, and psychotomimetic, inhibits N-methyl-d-aspartate (NMDA) receptors, while stimulating glutamate release and potentiating glutamate function through non-NMDA receptors such as α-amino-3-hydroxy-5-methylisoxazole-4-propionic acid (AMPA) receptors.^16^ Our study is based on preliminary human data from retrospective and non-randomised studies that suggested that anaesthesia with ketamine, compared with other drugs, improves reorientation and word recall after ECT.^15^ Ketamine has a rapid antidepressant effect after a single dose of intravenous ketamine,^17^ but evidence is unclear about improved symptomatic benefit when ketamine is combined with ECT.^17^, ^18^

In this study, we investigated whether ketamine, given as an adjunct to conventional anaesthetics, reduced cognitive dysfunction caused by ECT in patients with depression who had consented to receive ECT as part of their usual care.

## Methods

### Study design and participants

The ketamine-ECT study was a multicentre, parallel-group, randomised, double-blind, placebo-controlled, superiority trial of ketamine added to standard ECT during anaesthetic induction in patients with severe depression. The hypotheses and protocol have been published^19^ and are also available online.

Recruitment was from secondary care settings referring into 11 ECT suites in seven National Health Service (NHS) Trusts in the North of England (appendix). Recruited patients had a diagnosis of depression and had already consented to having ECT after having being referred for the study by their treating consultant psychiatrist. Inclusion criteria were a diagnosis of a unipolar or bipolar depressive episode defined as moderate or severe (DSM-IV criteria), age of at least 18 years, and the ability and inclination to provide written consent to participate in the study. They were also required to have a verbal IQ equivalent to 85 or greater, be sufficiently fluent in English to validly complete neuropsychological testing, and medically fit to receive ketamine (based on American Society of Anesthesiologists score of 1, 2, or stable 3 and the opinion of an anaesthetist). Exclusion criteria were detention under the Mental Health Act (1983, as amended in 2007); inability to give informed consent; a primary psychotic or schizoaffective disorder; current primary obsessive compulsive disorder, anorexia nervosa, history of drug or alcohol dependence (DSM-IV criteria); ECT in the past 3 months (amended from 6 months on May 22, 2013); known hypersensitivity or contraindication to ketamine, concomitant medications used for ECT, or excipients in these injections; evidence of organic brain disease including dementia or substantial medical illness affecting neuropsychological function; use of etomidate as an induction agent; pregnancy or not taking adequate contraception; breastfeeding; and a score of less than 24 on the Mini Mental State Examination (MMSE).

Ethical approval was granted by the North West-Liverpool East Research Ethics Committee (REC Ref No. 12/NW/0021) on Jan 25, 2012. Clinical Trial Authorisation was given by the Medicines and Healthcare products Regulatory Agency (23148/0004/001-0001). All participants gave written informed consent to participate. The study is registered with the International Standard Randomised Clinical Trial Number registry, number ISRCTN14689382, and with the EU Clinical Trials register, EudraCT number 2011-005476-41.

### Randomisation and masking

Patients were randomly assigned (1:1) to receive either ketamine or saline as anaesthetic for their ECT treatment. Randomisation was done by the Christie Hospital Clinical Trials Co-ordination Unit (CTU) by use of permuted block randomisation, which varied randomly between four and eight, and was stratified by NHS Trust. The randomisation code, held by the CTU, was provided to the local site pharmacies. At randomisation, the site research assistant telephoned the CTU with participant details and the CTU then contacted the local site pharmacy to prepare the investigational drug according to the randomisation code. Ampoules containing clear solutions of ketamine (10 mg/mL) or 0·9% sodium chloride solution (saline) were provided in secondary tamperproof trial packaging, while still retaining their original labelling. Both patients and assessment and ECT treatment teams were masked to treatment allocation, although the anaesthetists administering the study medication were not. The anaesthetist broke the seal away from the psychiatric ECT team, drew up the trial medication into a syringe, and disposed of the ampoule without revealing which drug was being administered. Researchers responsible for outcome assessment did not attend ECT sessions. To assess success of masking, patients and assessors were invited to complete a questionnaire to indicate suspected treatment group after four ECTs and at the end of the ECT course, and the results were sent directly to the CTU in a sealed envelope for data entry.

### Procedures

Standard ECT protocols based on the Royal College of Psychiatrists' *ECT Handbook*, 2nd edn^20^ were agreed between centres, with ECT treatments scheduled twice a week using Thymatron IV (Somatics, LLC, Lake Bluff, IL, USA) or Mecta Spectrum 5000 (Mecta Corp, Lake Oswego, OR, USA) devices depending on site. After pre-oxygenation with 100% oxygen, anaesthesia given for ECT treatment was propofol (or thiopental if clinically indicated) combined with the muscle relaxant suxamethonium and remained the same for all ECT treatments unless change was required for clinical reasons. Ketamine (0·5 mg/kg) or saline was given as a slow intravenous bolus directly before anaesthetic induction at each treatment. Electrode placement was standard bitemporal or right unilateral (D'Elia) placement.^20^ After confirmation of complete muscle relaxation (cessation of pedal muscle fasciculation), seizure was induced by giving patients constant-current brief pulse stimuli (0·5 ms pulse width, increased to 1·0 ms if clinically indicated). Treatment dose was 1·5 times seizure threshold for bitemporal electrode placement, and 4–6 times threshold for right unilateral electrode placement on the basis of rapid stimulus titration in the first ECT session. The stimulus parameters remained the same until after the fourth treatment unless requiring change for clinical reasons. ECT treatments were administered twice weekly to the end of the ECT treatment course, which was decided by each patient's treating clinical team. Standard operating procedures were used to identify criteria for an adequate seizure, for re-stimulation, and for ECT dosage adjustments to ensure adequate seizures and to optimise clinical response. Following the ECT course, patients were treated as usual by their clinical team; if a further course of ECT was started during the follow-up period they were withdrawn from the study.

Oral psychotropic medication was continued by the treating clinical team and not changed during the first four ECT treatments, and if possible, until the end of the course of ECT, unless required for safety or clinical reasons. The goal was to treat patients to remission (defined as Montgomery-Åsberg Depression Rating Scale [MADRS] ≤10),^2^ but the final decision to finish ECT treatment rested with the treating clinical team.

### Outcomes

The primary outcome was anterograde verbal memory measured by delayed recall on the Hopkins Verbal Learning Test-Revised (HVLT-R-DR)^21^ after four ECT treatments (±1 treatment); originally Controlled Oral Word Association Test (COWAT) category fluency and Autobiographical Memory Interview-Short Form (AMI-SF) had been included as primary outcomes, but these were not pursued, as discussed in the statistical analysis. Secondary neuropsychological assessments were HVLT-R-DR at the end of the course of ECT, and at 1 and 4 months of follow-up after ECT; at all timepoints, measurements were taken using other HVLT tests (learning, retention, recognition discrimination), COWAT, AMI-SF, Medical College of Georgia Complex Figure Test (MCGCFT), clinical digit span forwards and backwards, and Self-reported Global Self Evaluation of Memory (GSE-My). Reorientation was assessed 30 min after each ECT treatment (instrument references given in appendix). Secondary clinical efficacy measures were assessed at baseline, weekly during the ECT course, within 5 days of the final ECT, and at 1 and 4 months after the end of ECT, and consisted of MADRS,^22^ the Clinical Anxiety Scale (CAS), Clinical Global Impression (CGI), the 19-item Brief Psychiatric Rating Scale (BPRS; which included an elevated mood item), the Quick Inventory of Depressive Symptomatology—Self Report (QIDS-SR), and quality of life using the EuroQol 3-level version (EQ-5D-3L). We also calculated occurrence and time to remission (MADRS ≤10) and response (≥50% decrease in MADRS from baseline) during the ECT treatment course and noted the number of patients worsening during follow-up (defined as a MADRS increase of ≥4 points and CGI-severity increase of ≥1 point to CGI-severity ≥3 from the end of treatment assessment).

### Statistical analysis

We based the sample size on a moderate standardised effect size for the difference between ketamine and saline (0·5–0·6). We originally powered the study to detect a standardised effect size of 0·51 with 80% power, with a two-sided p value of 0·05 (after Bonferroni correction), for three primary cognitive outcome measures (HVLT-R-DR, COWAT category fluency, and AMI-SF). This required 80 patients per group assuming a 5% attrition rate after four ECT treatments. Following initial slow recruitment, the recruitment target was revised to 100 patients (50 patients per group) with the consent of the funder and Data Monitoring and Ethics Committee (DMEC), and a single primary outcome, the HVLT-R-DR specified. Assuming a 5% attrition for the primary outcome timepoint, this sample size gives 80% power to detect an effect size of 0·57, with a two-sided p value of 0·05.

The full statistical analysis plan was agreed with DMEC before study completion and before unmasking (appendix). Statistical analysis was based on a modified intention-to-treat population, defined as all patients who received the first ECT, depending on the availability of data. Missing efficacy data were filled in by pro-rating on the provision that at least 70% of items in the same scale had been completed; but was not used for neuropsychological outcomes. Neuropsychological assessments were linked to ECT treatments; mid-ECT (after 4±1 ECT) within 5 days of previous ECT treatment, and end of treatment (after last ECT) within 12 days of previous ECT treatment were deemed valid (appendix). This prespecified assessment window was broader than original protocol timings (3 and 5 days, respectively) because of clinical unpredictability of ECT treatment (eg, missed sessions, uncertainty about end of treatment), which hampered completing assessments within the original timeframes. We did a sensitivity analysis for the primary outcome measure limited to participants with completed assessments within the original protocol timings.

For each neuropsychological outcome a Gaussian repeated measures model with an unstructured covariance matrix was fitted by use of the mixed procedure in Stata, by use of all available data taking into account correlations between measures for each patient (this was a modification of the statistical analysis plan to avoid having to apply four models per outcome). On the basis of the observed mean time from baseline, 2, 6, 10, and 22 weeks were selected as the nominal time values in the repeated measures models. The treatment effect at each timepoint was assessed by the time by treatment interaction adjusting for prespecified covariates: baseline value, age at randomisation, sex, baseline degree of treatment resistance (Massachusetts General Hospital Scale [MGHS] score), and electrode placement (bitemporal or right unilateral). Only participants with at least one postbaseline assessment could therefore be included in the analysis. For the AMI-SF, we used scores measured at each timepoint rather than percentage of baseline recall (presented in the appendix). All repeated measures model analyses involved the use of robust standard errors and associated confidence intervals and p values (allowing for non-normality and constraints in the ranges of some of the cognitive outcomes).

We analysed acute efficacy data for the ECT treatment period over time using a random effects (random intercepts and slopes) analysis of covariance model with time in weeks since first ECT as a continuous variable with data provided until the end of treatment assessment. The fixed covariates were treatment, time by treatment interaction, and the same prespecified baseline characteristics as were used for the neuropsychological repeated measures analyses. For each model, two correlated random effects terms were included to capture between-subject variation in the intercept and slopes, assuming that people might improve at different rates. All models involved the use of robust standard errors and associated confidence intervals and p values. We did a sensitivity analysis for the MADRS using time since randomisation instead of from first ECT to investigate whether delay in starting ECT after randomisation affected the results. For remission and response binary variables, we analysed the frequency and cumulative frequency of first occurrence according to the week recorded using Kaplan-Meier plots and Cox analyses (adjusting for the same baseline variables as for continuous variables, including baseline MADRS). Time to event was defined as the difference between the first ECT date and the date of assessment, with last known efficacy assessment date used for censored patients. We compared the proportion of patients worsening at follow-up assessment (provided that patients had attended at least one follow-up timepoint) using Fisher's exact test.

We analysed statistical data using Stata Release 13 (Stata Statistical Software, StataCorp, TX, USA) and IBM SPSS Statistics 22·0 (IBM, Armonk, NY, USA).

### Role of the funding source

The funder of the study had no role in study design, data collection, data analysis, data interpretation, or writing of the report. The corresponding author had full access to all data in the study and made the decision to submit the report for publication.

## Results

628 patients received ECT in participating sites during the period of recruitment (early December, 2012, to mid-June, 2015). 202 (32%) of 628 patients were deemed potentially eligible from preliminary clinical and case note information, of whom 159 (79%) were invited to see the research team, 111 (55%) agreed to do so, and 79 (39%) were randomly assigned (1:1) to treatment (40 in the ketamine group *vs* 39 in the saline group; figure). Eligible patients were younger than those who were ineligible (mean age 57·9 [SD 13·6] *vs* 64·6 [SD 15·3], p<0·001) with an equal proportion being women (69·4% *vs* 66·0%). Included patients were younger than non-included potentially eligible patients (55·2 [SD 13·1] *vs* 59·8 [SD 13·7] years, p=0·02). The main reasons for non-progression were detention under the Mental Health Act (294 of 628, 47%), followed by impaired capacity in voluntary patients (44, 7%). 70 patients (89% of 79 randomly assigned to treatment) formed the modified intention-to-treat population (33 in the ketamine group *vs* 37 in the saline group). The exclusions to the modified intention-to-treat population included two patients in the ketamine group who had commenced ECT but were discovered retrospectively to meet exclusion criteria leading to ECT being discontinued (undiagnosed brain tumour, re-diagnosis of dementia). Exclusion from the modified intention-to-treat population occurred before analysis since their conditions were potential confounds for the cognitive outcome measures. Three patients, under-dosed with ketamine by 13–33% in error (two for the whole ECT course and one for only two doses), and one patient in the ketamine group who declined the study drug during the ECT course, were retained in the analysis.

At baseline, patients in the modified intention-to-treat population mostly had unipolar major depressive disorder with a severe current episode that had failed to respond to about four pharmacological treatments. The saline and ketamine groups were similar on demographics and clinical characteristics (table 1).

Most patients received propofol as the induction agent (table 2). Patients in the ketamine group received a 16% lower dose of propofol compared with the saline group since anaesthetists took ketamine into account in calculating the dose for anaesthesia.^24^ The two groups were similar with regard to ECT stimulus dose, seizure duration, and degree of reorientation at 30 min (table 2). Masking of treatment allocation was successful as assessed by questionnaire; patient guesses were correct in 28 (48%) of 58 patients who completed the questionnaire at mid-ECT and 30 (56%) of 54 patient guesses at the end of treatment, while 35 (56%) of 63 assessor guesses at mid-ECT and 28 (51%) of 55 assessor guesses at the end of treatment were correct, which did not differ significantly from chance.

The timing of valid neuropsychological assessments was similar in the two groups in terms of number of previous ECT treatments (mean 4·2 treatments [SD 0·6] in the ketamine group *vs* 4·0 [0·5] in the saline group at mid-ECT; 11·4 treatments [4·1] in the ketamine group *vs* 11·3 [4·5] in the saline group at end of treatment) and number of days between previous ECT session and assessment (2·2 days [1·2] in the ketamine group *vs* 1·8 [1·0] in the saline group at mid-ECT; 4·0 days [2·4] in the ketamine group *vs* 4·2 [2·4] in the saline group at end of treatment). The assessments done at 1 month (10·3 weeks [2·2] in the ketamine group *vs* 10·0 [2·7] in the saline group) and 4 months (21·9 weeks [3·4] in the ketamine group *vs* 21·8 [4·1] in the saline group) after the first ECT were done at a similar number of weeks.

For neuropsychological outcomes, there was no significant difference between groups on the HVLT-R-DR test at the primary outcome timepoint (mid-ECT) or at other timepoints (table 3, appendix). The sensitivity analysis excluding assessments 4 and 5 days after the last ECT at mid-ECT (n=4) and assessments 6–12 days after the last ECT at end of treatment (n=11) did not significantly change the results (appendix). The same pattern was seen for all secondary neuropsychological outcomes, with isolated significant advantage for the saline-treated group at mid-ECT for forward digit span and at end of treatment for HVLT-R recognition discrimination (table 3). At mid-ECT the standardised effect sizes (Cohen's d) were −0·13 (95% CI −0·54 to 0·27) for HVLT-R-DR, 0·01 (−0·37 to 0·38) for COWAT category fluency, and −0·07 (−0·33 to 0·19) for AMI-SF. For the same measures, effect sizes at end of treatment were −0·01 (−0·41 to 0·38) for HVLT-R-DR, −0·28 (−0·68 to 0·12) for COWAT category fluency, and −0·01 (−0·36 to 0·33) for AMI-SF. Subjective memory rated on the GSE-My did not differ between treatments with both groups rating ECT as causing a slight average worsening of memory, although self-assessment of current memory changed little through the ECT course (appendix).

There was no significant difference between the efficacy treatment slopes during the ECT treatment period (table 4, MADRS shown in the appendix). The marginal differences at specific timepoints for the MADRS that were calculated from the time by treatment interaction were similar: −2·2 (95% CI −5·6 to 1·3) at 2 weeks, −3·0 (−8·4 to 2·3) at 4 weeks, and −3·9 (−11·8 to 3·9) at 6 weeks. The marginal effect size at 2 weeks was −0·20 (−0·50 to 0·12) and at 6 weeks (based on SD at the end of treatment) −0·37 (−1·12 to 0·37) in favour of saline. A sensitivity analysis of the MADRS based on time since randomisation rather than first ECT showed similar results (appendix). In the ketamine group 13 (39%) of 33 patients reached remission (MADRS ≤10) during the acute treatment phase compared with 13 (35%) of 37 in the saline group; similarly, responses (MADRS ≥50% decrease from baseline) were 16 (49%) of 33 patients in the ketamine group and 22 (60%) of 37 in the saline group. On fitting a Cox model, the estimated hazard ratio [HR] for ketamine compared with saline was 1·16 (95% CI 0·51–2·64, p=0·73) in favour of remission in the ketamine group, a non-significant difference (appendix). The mean number of ECT treatments to achieve remission was 10·0 (SD 4·7) for the ketamine group and 7·0 (3·6) for the saline group. Applying a linear regression model, adjusting for the prespecified baseline covariates and baseline MADRS score, the mean number of ECT treatments to achieve remission for patients in the ketamine group was −0·83 (95% CI −4·9 to 3·2, p=0·67) greater than on saline, a non-significant difference.

In the ketamine group, four (16%) of 25 patients worsened (MADRS ≥4 point increase and CGI severity ≥3) during follow-up compared with eight (35%) of 23 in the saline group (Fisher's exact test p=0·19). One other patient met remission criteria by the end of follow-up in the ketamine group, and five more in the saline group; with 42·4% in the ketamine group and 48·6% in the saline group reaching remission by the end of study.

Seven serious adverse events occurred during the study (two in the ketamine group and five in the saline group) with no serious adverse reactions. Five serious adverse events involved worsening of depression (four with self-harm) and one each of chest pain and spontaneous seizures between ECT treatments (both in the saline group; table 5). In the ketamine group, 22 adverse events were reported in 15 (45%) of 33 patients compared with ten (27%) of 37 patients in the saline group, who had 13 adverse events, a non-significant difference. Three patients given ketamine had four adverse reactions that did not lead to treatment discontinuation. One participant had transient asymptomatic increased liver enzymes during ECT treatment, and two patients in the ketamine group experienced psychological effects after individual ECT treatments that did not recur, one of whom subsequently had vivid dreams.

## Discussion

This randomised trial tested the hypothesis that low-dose ketamine given alongside a course of ECT treatment would improve outcomes for depression. We found no evidence of benefit from adjunctive ketamine given at a dose of 0·5 mg/kg on cognitive and efficacy outcomes in patients being treated with ECT for depression. However, the lower than predicted recruitment means that small-to-moderate sized benefits and moderate-to-large sized harms of ketamine cannot be excluded. For the primary outcome at mid-ECT, an effect size benefit for ketamine of 0·3 or greater has been excluded with 95% confidence, and an effect size of 0·4 or greater excluded for other key outcomes; this is less than the moderate effect size originally proposed as clinically important. Two patients in the ketamine group had transient psychological effects following isolated ECT treatments that were probably related to ketamine, but there was no evidence of serious tolerability or safety problems with ketamine given at the dose provided in the study.

The rationale for the trial was the protective effect of ketamine on adverse cognitive effects in animal studies of electroconvulsive shock^25^ and from preliminary human data with ECT.^26^, ^27^, ^28^ Since designing this study, further small-to-medium studies (with 15–66 patients per treatment group) have produced inconsistent findings. A randomised trial^24^ using ultra-brief right unilateral ECT with similar neuropsychological tests to those in our study found no effect of ketamine (0·5 mg/kg) as an adjunct to thiopental. Three randomised trials have reported potential benefits on some of the outcomes used in this trial. Zhong and colleagues^29^ randomly assigned participants receiving ECT to receive either ketamine alone (0·8 mg/kg), ketamine (0·5 mg/kg) adjunctive to propofol (0·5 mg/kg), or propofol alone (0·8 mg/kg); the authors reported less impairment in frontal executive function only in participants who received ketamine alone, but no group differences were observed in verbal fluency, digit span, or the digit symbol test. Yoosefi and colleagues^30^ reported that ketamine (1–2 mg/kg) might prevent a decline in the MMSE after ECT compared with thiopental. The largest published randomised trial so far^31^ has reported that ketamine (0·3 mg/kg) as an adjunct to propofol significantly reduced the effect of ECT on a range of aspects of memory using a modified Chinese version of the Wechsler Memory Scale. By contrast, Rybakowski and colleagues,^32^ in a non-randomised prospective study, found that ketamine (1–1·5 mg/kg) as an adjunct to thiopental at alternate ECT sessions worsened verbal memory and had a trend to worsen letter fluency, but did not affect executive function, visual memory, or digit span compared with thiopental alone. Similarly, results for reorientation after ECT are conflicting; one randomised trial found adjunctive ketamine (0·3 mg/kg) led to more rapid re-orientation compared with propofol alone,^33^ while a randomised crossover study with ketamine (1 mg/kg) delayed reorientation compared with methohexital.^34^ Interpretation of the literature is made difficult by small sample sizes and differences in methods used by different groups. Our results appear to directly contrast with those of Chen and colleagues;^31^ however, differences in methods include the use of a 1 ms pulse width stimulus, ECT given three times a week, a lower ketamine dose, and a Chinese population that had considerably greater memory impairment from ECT than in our study. It is uncertain how far their results can be extrapolated to ECT as given in the UK.

The efficacy evidence is also variable. A meta-analysis that collated data from five randomised trials found no difference in efficacy between patients treated with ketamine or placebo;^18^ by contrast, a previous meta-analysis included four of these studies and reported a significant benefit for ketamine given with ECT early in the treatment course.^17^ We found a further six randomised trials not included in these meta-analyses. Four found no greater efficacy with ketamine,^31^, ^33^, ^35^, ^36^ and two reported greater efficacy in the ketamine-treated group,^29^, ^37^ although of these two studies, there is uncertainty about the statistical robustness in one^37^ and the plausibility of the clinical responses seen in the other.^29^ The study reported here found a small numerical advantage in the saline-treated group with the 95% confidence interval excluding more than small-to-moderate advantage with ketamine treatment.

Some studies have suggested that ketamine could accelerate the clinical response to ECT,^17^, ^24^, ^29^, ^30^, ^31^, ^38^ but we did not find any evidence for this and, on average, participants treated with ketamine achieved remission later than those on saline, although this observation was not significant. This is in sharp contrast to the rapid antidepressant effect within hours or days reported when ketamine is given alone.^17^ Explanations for this apparent discrepancy could include ECT blocking the effects of ketamine (possibly suggesting involvement in a common pathway), the requirement of a slow ketamine infusion rather than a bolus for the antidepressant effect, or requiring a subjective experience of ketamine's dissociative effects.

The twice-weekly, predominantly bitemporal ECT used in our study with a stimulus pulse width of 0·5 ms did not appear to cause a substantial reduction in objective, or self-reported, cognitive function, and the decrease in AMI-SF values over time is similar to that seen in healthy volunteers.^39^ Bitemporal ECT is the standard electrode placement in the UK^5^ by use of a 0·5 ms pulse width. It has been suggested that a 0·5 ms pulse width causes less cognitive impairment than the 1·0–1·5 ms pulse widths predominantly reported in the literature,^40^ which might explain the modest cognitive effects we found in this Article, but this finding needs to be studied systematically. Remission rates in our study were relatively low (37%) compared with about half of patients remitting in Scotland during 2014.^5^ A possible explanation is that remission rates are lower in patients without psychosis^5^ and with treatment resistance^4^ as was predominantly found in our patients.

Few studies have followed up patients after the end of ECT; one study found an efficacy advantage to the patients treated with ketamine 1 week after ECT but not after 1 month.^24^ We found no difference in cognitive or efficacy outcomes 4 months after ECT, although the dropout rate restricts interpretation, since those patients that discontinued treatment might be affected by clinical factors such as relapse or further ECT.

We did not find any major safety or tolerability problems attributable to ketamine, although two (6%) of 33 patients treated with ketamine had transient psychological reactions. Some studies using ketamine alone, or at higher doses than used in this study, have found it to be associated with slower reorientation and more adverse events than control conditions.^34^, ^35^, ^41^ These effects appear to be attenuated when ketamine is combined with propofol,^41^ indicating that the dose and context of ketamine administration is important when assessing its safety and tolerability with ECT.

Our study has a number of limitations. The most important is that the sample size was smaller than planned, which resulted in a low power so that we cannot exclude either a small to moderate sized benefit or moderate harm from treatment with adjunctive ketamine. Although we did not have differential dropout between treatment groups, over 40% of the patient population had dropped out by the 1-month follow-up and almost half by 4-month follow-up. We therefore have limited ability to say what happened to patients after the end of treatment timepoint. The clinical realities of treatment made strictly timed assessments difficult, but there were no systematic differences seen in assessment timing or delay between the treatment groups, and sensitivity analyses with stricter timings did not alter the results. The included patients were not fully representative of the total population of patients who receive ECT; in particular, they were younger and, based on reasons for exclusion, likely to be less cognitively compromised and less severely ill than non-eligible patients so that generalisation to these more severely affected patients cannot be assumed; however, trials in these patients would present considerable ethical difficulties. Finally, our results are based on one dose and method of administering ketamine with ECT, and it is possible that results could be different with other doses of ketamine or in the context of different ECT regimens or patient populations.

The place of ketamine in combination with ECT has not been finally defined, but our study suggests that there is no beneficial effect when it is given at a standard dose of 0·5 mg during ECT as it is routinely given in the UK. Future research using small to moderate sample sizes is likely to be unhelpful in further resolving the question, and only a sufficiently large future study would be able to address this uncertainty. There are considerable practical difficulties in doing multicentre ECT research and the uncertainty about the optimum dose and method for administering ketamine with ECT is an important challenge for the design of a definitive study.

## Figures and Tables

**Figure:**
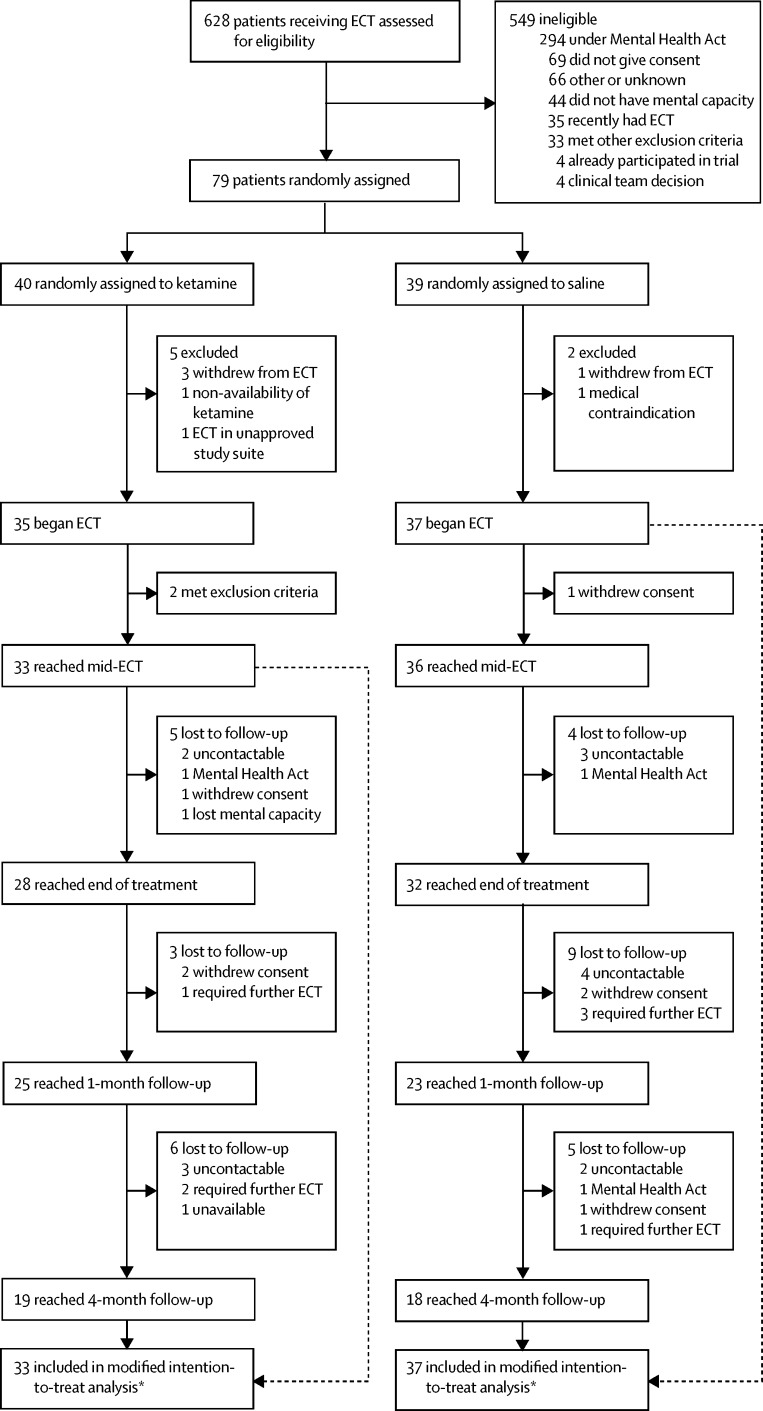
Trial profile *Modified intention-to-treat was defined as all patients randomised to treatment who received a first ECT. Analysis required one or more valid postbaseline assessments: numbers analysed were 32–33 in the ketamine group and 35–36 in the saline group depending on outcome measure.

**Table 1:** Baseline demographics and clinical characteristics of participants

		**Ketamine group (n=33)**	**Saline group (n=37)**
Mean age, years		52·5 (11·9)	56·4 (12·4)
Sex, female		22 (67%)	22 (60%)
Mean years in full-time education		13·7 (4·0)	13·5 (3·2)
Ethnicity white		31 (94%)	35 (95%)
Married or current partner		13 (39%)	19 (51%)
Mean IQ		105·1 (11·3)	109·9 (11·0)
MMSE		28·8 (2·0)	29·0 (1·2)
Illness characteristics			
	Inpatient	11 (33%)	21 (57%)
	Bipolar disorder	4 (12%)	7 (19%)
	Age of onset (years)	29·6 (14·0)	32·2 (17·0)
	Number of depressive episodes (lifetime)	4·9 (4·4)	5·3 (5·2)
	Number of hypomanic/manic episodes	0·3 (1·0)	0·5 (1·8)
	Previous ECT	18 (55%)	16 (43%)
	Family history of depression	23 (70%)	18 (49%)
	Family history of bipolar disorder	2 (6%)	10 (27%)
Current depressive episode			
	MADRS score	31·8 (7·4)	35·2 (8·4)
	Melancholia no psychosis	26 (79%)	25 (68%)
	Psychosis ± melancholia	3 (9%)	8 (22%)
	Median duration, months	14 (7–38)	8 (3·5–20·5)
	MGHS score	4·8 (2·6)	4·0 (3·4)
Comorbidity			
	Anxiety disorder or secondary OCD	16 (48%)	18 (49%)
	Other psychiatric disorder	1 (3%)	0 (0%)
	Medical illness	12 (36%)	9 (24%)
Physical signs			
	Weight, kg	82·2 (17·1)	77·2 (17·7)
	BMI, kg/m^2^	29·3 (5·8)	28·8 (6·7)
	Systolic blood pressure, mmHg	132·9 (18·0)	126·1 (17·2)
Psychotropic medication			
	SSRI	10 (30%)	14 (38%)
	SNRI	16 (48%)	15 (41%)
	TCA	4 (12%)	8 (22%)
	MAOI	0 (0)	1 (3%)
	Other antidepressant	14 (42%)	10 (27%)
	Antipsychotic	18 (55%)	26 (70%)
	Lithium	7 (21%)	5 (14%)
	Antiepileptic mood stabiliser	5 (15%)	7 (19%)
	Hypnotic/anxiolytic	17 (52%)	21 (57%)

Data are mean (SD), n (%), or median (IQR). BMI=body-mass index. IQ=intelligence quotient. MAOI=monoamine oxidase inhibitor. MGHS=Massachusetts General Hospital Scale. MMSE=Mini Mental State Examination. OCD=obsessive compulsive disorder. SNRI=serotonin and noradrenaline reuptake inhibitor. SSRI=selective serotonin reuptake inhibitor. TCA=tricyclic antidepressant.

**Table 2:** ECT treatment variables and re-orientation in the two treatment groups

	**Ketamine group (n=33)**	**Saline group (n=37)**	**p value**
**Dose of drugs for induction**			
Propofol (mg)	127·1 (33·9)	151·4 (43·6)	0·01
Thiopental (mg)*	334·9 (53·4)	150	..
Suxamethonium (mg)	49·8 (15·0)	50·8 (14·3)	0·77
Ketamine or saline (mg)†	40·8 (8·1)	38·8 (8·1)	0·32
**ECT-related variables**			
Bilateral ECT at initiation‡	28 (85%)	34 (92%)	0·46
Bilateral ECT at mid-ECT‡	28 (83%)	32 (89%)	0·26
Stimulus dose (mC)	306·0 (213·6)	276·5 (155·6)	0·51
Motor seizure duration (s)	15·2 (5·6)	14·9 (5·2)	0·83
EEG seizure duration (s)	24·4 (10·4)	26·2 (7·8)	0·42
**Reorientation after seizure**			
Items correct at 30 min (of 5)	4·65 (0·50)	4·69 (0·34)	0·69
Proportion of sessions each participant was reoriented at 30 min§	0·93 (0·14)	0·94 (0·11)	0·75

Data are mean (SD) or n (%). Data exclude first ECT session, which was a titration session, and use last stimulation in case of re-stimulation within a treatment session. Reorientation was assessed by use of five questions (name, place, day of the week, age, and birthday).^23^ EEG=electroencephalogram.

* Two patients from the ketamine group received thiopental from initiation, a further two patients from the ketamine group and one from the saline group switched to thiopental from propofol during the ECT course.

† Saline dose is mg equivalent of ketamine. One patient in the ketamine group withdrew consent to the study drug after the first ECT but continued in the trial.

‡ Over the full course of ECT two patients treated with saline and four treated with ketamine switched from unilateral to bilateral ECT, and one patient from each group switched from bilateral to unilateral ECT.

§ Being reoriented was defined as correctly answering at least 4 of 5 orientation questions.

**Table 3:** Neuropsychological task outcomes in participants randomised to saline or ketamine

		**Ketamine group**		**Saline group**		**Repeated measures analysis**	
		Mean score (SD)	n	Mean score (SD)	n	Difference ketamine–saline adjusted for baseline (95% CI)	p value
**HVLT-R**							
Delayed recall							
	Baseline	6·12 (2·69)	33	5·86 (3·63)	37	..	..
	Mid-ECT*	5·17 (2·92)	29	5·54 (3·42)	35	−0·43 (−1·73 to 0·87)	0·51
	End of treatment	5·69 (2·80)	26	5·44 (3·18)	32	−0·04 (−1·22 to 1·13)	0·94
	1-month follow-up	6·70 (2·67)	23	7·26 (2·63)	23	−0·53 (−1·66 to 0·60)	0·36
	4-month follow-up	6·63 (3·17)	19	8·11 (2·83)	18	−1·40 (−2·91 to 0·12)	0·07
Total learning							
	Baseline	20·0 (4·1)	33	20·8 (6·7)	37	..	..
	Mid-ECT*	20·2 (5·8)	29	21·0 (5·4)	36	−0·11 (−2·04 to 1·81)	0·91
	End of treatment	19·8 (4·9)	26	21·2 (5·6)	32	−0·96 (−2·97 to 1·06)	0·35
	1-month follow-up	22·3 (4·7)	23	23·5 (5·6)	23	−0·50 (−2·54 to 1·54)	0·63
	4-month follow-up	21·6 (5·0)	19	24·1 (6·1)	18	−1·12 (−3·68 to 1·44)	0·39
Retention							
	Baseline	73·3 (27·8)	33	65·2 (32·9)	37	..	..
	Mid-ECT*	58·6 (25·0)	29	62·4 (31·8)	35	−5·12 (−18·0 to 7·8)	0·44
	End of treatment	69·4 (26·6)	26	58·7 (26·4)	32	6·97 (−4·88 to 18·8)	0·25
	1-month follow-up	73·3 (24·0)	23	77·1 (16·1)	23	−3·22 (−13·3 to 6·9)	0·53
	4-month follow-up	74·2 (23·3)	19	85·5 (18·7)	18	−11·97 (−24·0 to 0·10)	0·052
Recognition discrimination							
	Baseline	9·45 (2·05)	33	8·16 (3·48)	37	..	..
	Mid-ECT*	8·48 (2·81)	29	8·54 (2·84)	35	−0·64 (−1·72 to 0·44)	0·25
	End of treatment	8·58 (2·90)	26	9·66 (2·10)	32	−1·59 (−2·76 to −0·42)	0·008
	1-month follow-up	9·52 (2·33)	23	9·52 (2·33)	23	−0·59 (−1·61 to 0·43)	0·25
	4-month follow-up	10·42 (1·43)	19	9·56 (2·97)	18	0·51 (−0·78 to 1·80)	0·44
**COWAT**							
Letter fluency							
	Baseline	33·8 (13·1)	33	36·1 (14·3)	37	..	..
	Mid-ECT*	31·5 (12·9)	29	36·1 (13·2)	36	−1·82 (−5·86 to 2·23)	0·38
	End of treatment	33·0 (13·4)	26	34·2 (13·5)	32	−1·01 (−5·57 to 3·54)	0·66
	1-month follow-up	35·6 (13·4)	23	39·5 (14·1)	23	−1·52 (−5·74 to 2·70)	0·48
	4-month follow-up	37·6 (13·2)	19	38·6 (10·7)	18	−0·18 (−5·17 to 4·80)	0·94
Category fluency							
	Baseline	15·8 (5·5)	33	16·8 (5·3)	37	..	..
	Mid-ECT*	15·9 (5·3)	29	16·4 (4·2)	36	0·03 (−1·73 to 1·78)	0·97
	End of treatment	14·3 (5·1)	26	15·8 (4·2)	32	−1·29 (−3·15 to 0·57)	0·17
	1-month follow-up	16·5 (5·6)	23	17·1 (4·1)	23	−0·23 (−2·43 to 1·96)	0·84
	4-month follow-up	17·8 (4·6)	19	18·1 (5·3)	18	−0·35 (−2·90 to 2·21)	0·79
**AMI-SF**							
	Baseline	45·5 (9·2)	33	44·2 (10·3)	37	..	..
	Mid-ECT*	39·3 (9·0)	29	38·0 (10·0)	36	−0·67 (−3·16 to 1·81)	0·60
	End of treatment	34·7 (9·8)	25	34·8 (10·5)	32	−0·11 (−3·63 to 3·41)	0·95
	1-month follow-up	35·1 (10·0)	23	35·4 (10·4)	23	−0·46 (−3·91 to 3·00)	0·80
	4-month follow-up	37·4 (10·1)	19	38·9 (8·4)	16	−0·70 (−3·93 to 2·54)	0·67
**MCGCFT**							
Copy							
	Baseline	34·6 (2·4)	33	33·4 (4·3)	37	..	..
	Mid-ECT*	33·9 (4·0)	29	34·1 (3·2)	36	−0·66 (−2·27 to 0·95)	0·42
	End of treatment	34·6 (2·0)	26	34·4 (2·4)	32	−0·54 (−1·66 to 0·57)	0·34
	1-month follow-up	35·3 (1·2)	23	34·4 (3·0)	23	−0·41 (−1·61 to 0·78)	0·50
	4-month follow-up	35·2 (1·1)	19	35·0 (1·1)	18	−0·49 (−1·32 to 0·34)	0·25
Immediate recall							
	Baseline	19·0 (8·8)	33	17·9 (7·6)	37	..	..
	Mid-ECT*	18·6 (8·6)	29	18·4 (7·2)	36	−0·35 (−3·09 to 2·39)	0·80
	End of treatment	19·0 (7·4)	26	16·6 (6·2)	32	0·61 (−1·77 to 3·00)	0·61
	1-month follow-up	19·5 (6·0)	23	19·7 (6·7)	23	−1·96 (−4·43 to 0·50)	0·12
	4-month follow-up	23·8 (8·1)	19	21·5 (6·4)	18	−0·27 (−3·05 to 3·58)	0·88
Delayed recall							
	Baseline	18·9 (7·7)	33	17·6 (6·9)	36	..	..
	Mid-ECT*	17·7 (8·2)	29	17·7 (7·5)	35	−0·77 (−3·64 to 2·10)	0·60
	End of treatment	17·6 (5·9)	25	15·3 (5·3)	32	0·21 (−1·94 to 2·36)	0·85
	1-month follow-up	18·9 (6·4)	22	19·0 (6·5)	23	−2·32 (−5·00 to 0·37)	0·09
	4-month follow-up	22·9 (9·7)	19	20·8 (6·8)	18	0·29 (−3·79 to 4·36)	0·89
**Digit span**							
Forward							
	Baseline	5·70 (1·07)	33	5·84 (1·21)	37	..	..
	Mid-ECT*	5·48 (0·95)	29	6·00 (1·04)	36	−0·46 (−0·81 to −0·11)	0·009
	End of treatment	5·88 (1·34)	26	5·84 (1·02)	32	0·003 (−0·49 to 0·50)	0·99
	1-month follow-up	5·91 (1·04)	23	5·83 (1·11)	23	0·002 (−0·46 to 0·46)	0·99
	4-month follow-up	5·68 (1·20)	19	5·72 (0·89)	18	−0·18 (−0·64 to 0·27)	0·43
Backwards							
	Baseline	3·64 (1·17)	33	3·95 (1·10)	37	..	..
	Mid-ECT*	3·86 (1·38)	29	3·94 (1·01)	36	0·17 (−0·25 to 0·59)	0·42
	End of treatment	3·88 (1·21)	26	3·97 (0·97)	32	−0·04 (−0·50 to 0·42)	0·87
	1-month follow-up	3·87 (1·25)	23	4·13 (1·29)	23	−0·14 (−0·70 to 0·43)	0·63
	4-month follow-up	3·89 (1·05)	19	4·06 (1·06)	18	−0·08 (−0·65 to 0·50)	0·80

Modified intention-to-treat repeated measures analysis was adjusted for age at randomisation, sex, baseline degree of treatment resistance, electrode placement (bilateral or unilateral), and baseline value of each outcome. Negative values favour saline. AMI-SF=Autobiographical Memory Interview-Short Form. COWAT=Controlled Oral Word Association Test. HVLT-R=Hopkins Verbal Learning Test-Revised. MCGCFT=Medical College of Georgia Complex Figure Test.

* Mid-ECT for these were approximately 2 weeks after baseline, but varied between patients.

**Table 4:** Efficacy outcomes in participants randomised to ketamine or saline

	**Ketamine group**		**Saline group**		**Treatment effect**	
	Mean score (SD)	n	Mean score (SD)	n	Estimated difference in slopes (95% CI)	p value
**MADRS**						
Baseline	31·8 (7·4)	33	35·2 (8·4)	37	..	..
Mid-ECT*	25·4 (9·8)	31	25·9 (12·4)	33	..	..
End of treatment	17·2 (11·6)	27	15·0 (10·4)	32	..	..
Ketamine *vs* saline from baseline up to end of treatment	..	..	..	..	−0·44 (−1·91 to 1·03)	0·56
1-month follow-up	16·8 (13·6)	24	14·8 (11·4)	23	..	..
4-month follow-up	18·0 (13·3)	19	13·5 (13·9)	18	..	..
**CAS**						
Baseline	7·79 (4·53)	33	9·16 (5·23)	37	..	..
Mid-ECT*	7·39 (5·29)	31	7·48 (5·37)	33	..	..
End of treatment	5·44 (4·04)	27	5·16 (4·34)	32	..	..
Ketamine *vs* saline from baseline up to end of treatment	..	..	..	..	−0·02 (−0·42 to 0·38)	0·93
1-month follow-up	4·96 (4·62)	24	4·43 (·89)	23	..	..
4-month follow-up	4·95 (4·73)	19	3·67 (4·92)	18	..	..
**BPRS**						
Baseline	37·3 (5·4)	33	40·0 (8·6)	37	..	..
Mid-ECT*	35·1 (6·9)	31	35·4 (8·5)	33	..	..
End of treatment	29·6 (6·9)	26	28·3 (6·1)	32	..	..
Ketamine *vs* saline from baseline up to end of treatment	..	..	..	..	−0·02 (−0·73 to 0·70)	0·97
1-month follow-up	28·8 (7·8)	24	27·6 (6·4)	23	..	..
4-month follow-up	30·3 (8·3)	19	27·1 (8·3)	18	..	..
**CGI-Severity**						
Baseline	5·03 (0·85)	33	5·30 (0·97)	37	..	..
Mid-ECT*	4·48 (1·12)	31	4·24 (1·35)	33	..	..
End of treatment	3·33 (1·33)	27	2·88 (1·24)	32	..	..
Ketamine *vs* saline from baseline up to end of treatment	..	..	..	..	−0·03 (−0·18 to 0·13)	0·73
1-month follow-up	2·79 (1·56)	24	2·57 (1·27)	23	..	..
4-month follow-up	3·00 (1·60)	19	2·17 (1·34)	18	..	..
**CGI-Improvement**						
Mid-ECT*	3·03 (1·02)	31	3·00 (1·09)	33	..	..
End of treatment	2·52 (1·31)	27	2·25 (1·11)	32	..	..
Ketamine *vs* saline from baseline up to end of treatment	..	..	..	..	0·03 (−0·11 to 0·17)	0·69
1-month follow-up	2·13 (1·26)	24	2·00 (1·04)	23	..	..
4-month follow-up	2·42 (1·35)	19	2·11 (1·41)	18	..	..
**QIDS-SR**						
Baseline	17·9 (4·9)	33	20·0 (3·9)	37	..	..
Mid-ECT*	14·1 (5·6)	31	16·0 (5·9)	33	..	..
End of treatment	11·9 (6·2)	27	11·0 (5·8)	32	..	..
Ketamine *vs* saline from baseline up to end of treatment	..	..	..	..	−0·30 (−0·88 to 0·29)	0·32
1-month follow-up	12·0 (7·5)	24	10·1 (6·2)	23	..	..
4-month follow-up	12·5 (7·7)	19	9·4 (7·5)	18	..	..
**EQ-5D-3L index**						
Baseline	0·35 (0·27)	33	0·35 (0·28)	37	..	..
Mid-ECT*	0·55 (0·28)	31	0·44 (0·38)	33	..	..
End of treatment	0·66 (0·26)	27	0·70 (0·27)	32	..	..
Ketamine *vs* saline from baseline up to end of treatment	..	..	..	..	0·01 (−0·01 to 0·03)	0·43
1-month follow-up	0·67 (0·33)	24	0·71 (0·29)	23	..	..
4-month follow-up	0·60 (0·33)	19	0·71 (0·34)	18	..	..
**EQ-5D-3L VAS**						
Baseline	31·9 (14·6)	33	24·2 (16·9)	37	..	..
Mid-ECT*	42·0 (18·6)	31	33·6 (22·2)	33	..	..
End of treatment	51·4 (22·3)	26	52·2 (21·9)	32	..	..
Ketamine *vs* saline from baseline up to end of treatment	..	..	..	..	1·16 (−1·09 to 3·41)	0·31
1-month follow-up	53·0 (26·4)	24	54·8 (30·2)	23	..	..
4-month follow-up	46·2 (24·6)	19	62·4 (31·1)	18	..	..

Values are means (SD) and n for valid assessments at each timepoint, and estimated difference in slopes (95% CI) for modified intention-to-treat treatment linear random effects analysis during ECT treatment alone adjusting for age at randomisation, sex, baseline degree of treatment resistance, electrode placement (bilateral or unilateral), and baseline value. Negative values favour saline. BPRS=Brief Psychiatric Rating Scale (18-item). CAS=Clinical Anxiety Scale (6-item). CGI=Clinical Global Impression. EQ-5D-3L (VAS)=EuroQol 3 level version (Visual Analogue Scale). MADRS=Montgomery-Åsberg Depression Rating Scale. QIDS-SR=Quick Inventory of Depression Symptomatology-Self Report.

* Mid-ECT for these outcomes were defined as 2 weeks after baseline.

**Table 5:** Details of adverse events or reactions with a frequency of greater than 5%

	**Ketamine group (n=33)**	**Saline group (n=37)**
Infections and infestations	3 (9%)	0
Musculoskeletal and connective tissue disorders	2 (6%)	0
Nervous system disorders	2 (6%)	1 (3%)
Psychiatric disorders	5 (15%)	7 (19%)
Skin and subcutaneous tissue disorders	2 (6%)	0

Data are n (%). Patients experiencing more than one occurrence of an adverse event in a system organ class (as defined in the Medical Dictionary for Regulatory Activities [MedDRA]) are counted once. Values include serious adverse events and adverse reactions.
