# Supplementary material for: Ketamine augmentation of electroconvulsive therapy to improve neuropsychological and clinical outcomes in depression (Ketamine-ECT): a multicentre, double-blind, randomised, parallel-group, superiority trial
Source: Lancet Psychiatry. 2017 May;4(5):365–77. doi: 10.1016/S2215-0366(17)30077-9 (PMC5406618; doi:10.1016/S2215-0366(17)30077-9)
Supplement: Supplementary appendix [file mmc1.pdf]

THE LANCET
Psychiatry

Supplementary appendix

This appendix formed part of the original submission and has been peer reviewed. We post it as supplied by the authors.

Supplement to: Anderson IM, Blamire A, Branton T, et al. Ketamine augmentation of electroconvulsive therapy to improve neuropsychological and clinical outcomes in depression (Ketamine-ECT): a multicentre, double-blind, randomised, parallel-group, superiority trial. *Lancet Psychiatry* 2017; published online March 27. http://dx.doi.org/10.1016/S2215-0366(17)30077-9.

Randomised-controlled trial of ketamine augmentation of ECT to improve neuropsychological and clinical outcomes in depression (Ketamine-ECT Study)

Anderson IM et al.

Supplementary Appendix

Ketamine-ECT study team

Members of the team not listed as primary authors were:
Claire Blakeley, Katherine Crosby, Aisha Perkis, Graham Spencer, Liam Trevithick, Amanda Watson, Francesca Williams, Audrey Williamson.

Support given to the study

The following made important contributions to the study: Moruf Adelekan, Mark Appleton, Bev Austin, Victoria Baron, Hannah Bayes, Suzy Bourke, Vanda Brack, Jackie Brammer, Jonathan Burden, Karen Butler, Vinod Chaugule, Anthony Cleare, Richard Cree, Michael Dixon, Beatriz Duran, Ali Ford-Brown, Lloyd Gregory, John Green, Louise Golightly, Sharon Grieve, Matthew Haggarty, Lewis Halpin, Guy Harvey, Kathryn Hayes, Nicola Hermitage, Stephen Holgate, Keith Holt, Graham Hough, Jennie Hunter, Versha Jhinghan, Coral Jones, Farzana Kausir, Janet Kennedy, Chintaharan Kotur, Sean Lennon, Maureen Longstaff, Diane Lyons, Celia Marshall, Andy Mee, Kirsty Melia, Jane Newby, Apsara Panikkar, Susanna Piggott, David Ralph, Ram Singh, Debbie Sutton, Albert Swana, Tim Ramsay, Sharron Robinson, Diane Ruddy, Darren Rusk, David Ryder, Elankathir Selvaraasan, Amanda Spencer, Maxine Syme, Andrew Syndercombe, Emma Taylor, Alan Thomas, Aparna Trivedi, Seema Varshney, Rachel Ward, Stuart Watson, Rhiannon Whitaker, Chris White, Carolyn Whitley, Judith Wilkes, Kate Williams, Jan Wood. We would also like to express thanks to the Local Clinical Research Networks: East Midlands, Greater Manchester, North East and North Cumbria, Yorkshire and Humber. Essential support and guidance was provided by the Christie Hospital (now Manchester Academic Health Science Centre) Clinical Trials Co-ordination Unit (Anne Bowers, Gemma Darby, Claire Goldrick, Simon Williams), the Service User Group (organiser Tim Rawcliffe), Trial Steering Committee (chair David Baldwin) and Data Monitoring and Ethics Committee (chair Keith Matthews).

NHS Trusts involved in trial

Derbyshire Healthcare NHS Foundation Trust, Lancashire Care NHS Foundation Trust, Leeds and York Partnership NHS Foundation Trust, Manchester Mental Health and Social Care Trust (now part of Greater Manchester Mental Health NHS Foundation Trust), Northumberland Tyne and Wear NHS Foundation Trust, Pennine Care NHS Foundation Trust and Tees, Esk and Wear Valleys NHS Foundation Trust.

Assessment references

Diagnostic and Statistical Manual of Mental Disorders, 4th Edition (DSM-IV) criteria.[1]
Mini Mental State Examination (MMSE).[2]
Controlled Oral Word Association Test (COWAT).[3]
Autobiographical Memory Interview - short form (AMI-SF).[4]
Medical College of Georgia Complex Figure Test (MCGCFT).[5]

Clinical digit span forwards and backwards.[6]
Self-reported Global Self Evaluation of Memory (GSE-My). [7]
Reorientation 30 min after each ECT treatment.[8]
Clinical Anxiety Scale (CAS).[9]
Clinical Global Impression (CGI).[10]
19-item Brief Psychiatric Rating Scale (BPRS).[11]
Quick Inventory of Depressive Symptomatology - Self Report (QIDS-SR).[12]
EuroQol 3 level version (EQ-5D-3L).[13]

1. American Psychiatric Association. *Diagnostic and Statistical Manual of Mental Disorders, Fourth Edition, Text Revision (DSM-IV-TR)*. Arlington, VA: American Psychiatric Association. 2000.
2. Folstein MF, Folstein SE, McHugh PR. 'Mini-Mental State': a practical method for grading the cognitive state of patients for the clinician. *Journal of Psychiatric Research.* 1975, 12: 189-198.
3. Benton LA, Hamsher K, Sivan AB. *Controlled Oral Word Association Test. Multilingual Aphasia Examination*. Iowa City: AJA. 1994.
4. McElhiney M, Moody B, Sackeim H. *The Autobiographical Memory Interview – Short Form.* New York: New York State Psychiatric Institute. 1997.
5. Meador KJ, Moore EE, Nichols ME, Abney OL, Taylor HS, Zamrini EY et al. The role of cholinergic systems in visuospatial processing and memory. *J Clin Exp Neuropsychol.* 1993, 15: 832-842.
6. Wechsler D. *Wechsler Adult Intelligence Scale-Revised.* New York: The Psychological Corporation. 1981.
7. Berman RM, Prudic J, Brakemeier EL, Olfson M, Sackeim HA. Subjective evaluation of the therapeutic and cognitive effects of electroconvulsive therapy. *Brain Stimul.* 2008, 1: 16-26.
8. Sobin C, Sackeim HA, Prudic J, Devanand DP, Moody BJ, McElhiney MC. Predictors of retrograde amnesia following ECT. *Am J Psychiatry.* 1995, 152: 995-1001.
9. Snaith RP, Baugh SJ, Clayden AD, Husain A, Sipple MA. The Clinical Anxiety Scale: an instrument derived from the Hamilton Anxiety Scale. *Br J Psychiatry.* 1982, 141: 518-523.
10. Guy W. *ECDEU assessment manual for psychopharmacology, revised. US Department of Health, Education and Welfare publication (ADM)*. Rockville Md: National Institute of Mental Health. 1976.
11. Overall JE, Gorman DR. The Brief Psychiatric Rating Scale. *Psychological Reports.* 2011, 10: 799-812.
12. Rush AJ, Trivedi MH, Ibrahim HM, Carmody TJ, Arnow B, Klein DN et al. The 16-Item Quick Inventory of Depressive Symptomatology (QIDS), clinician rating (QIDS-C), and self-report (QIDS-SR): a psychometric evaluation in patients with chronic major depression. *Biol Psychiatry.* 2003, 54: 573-583.
13. The EuroQol Group. EuroQol-a new facility for the measurement of health-related quality of life. *Health Policy.* 1990, 16: 199-208.

Statistical analysis plan (clinical outcomes)

Version 1.1 (27/10/2015) Agreed by Data Monitoring and Ethic Committee (Chair Prof. Keith Matthews) and approved by Trial Steering Committee (Chair Prof. David Baldwin)

Brief description of the trial

The trial aims to determine whether ketamine improves cognitive outcomes after ECT. The main hypothesis is that ketamine, compared with saline, treatment will reduce ECT-induced cognitive impairments in anterograde verbal memory after the mid-course of acute ECT treatment. The main secondary hypotheses are ketamine, compared with saline, treatment will reduce ECT-induced cognitive impairments in autobiographical memory and verbal fluency after the mid-course of acute ECT treatment. The subsidiary hypotheses are that ketamine, compared with saline, treatment will reduce ECT-induced cognitive impairments at the end of acute treatment with ECT, and speed the improvement in symptoms of depression.

Study design

Randomised, placebo-controlled, parallel study with blind assessment

Trial treatments

ECT treatments are scheduled twice weekly. In a 1:1 ratio subjects will receive either intravenous ketamine 0.5mg/kg or placebo as part of the anaesthetic each time ECT is administered. The goal will be to treat patients to remission (standard Montgomery Asberg Depression Rating Scale, MADRS ≤10) in accordance with NICE guidelines.

Randomisation procedure

Patients will be randomised in a 1:1 ratio to ketamine or saline following registration and before the first ECT using permuted block randomisation (varying blocks randomly from 4 to 8), stratified by inclusion by Trust for those not undergoing MR imaging, and by scanner site (Manchester or Newcastle) for those who are receiving MR imaging. The randomisation code will be generated by the Christie CTU and provided to the local pharmacies for drug preparation when a patient is recruited. For safety reasons the anaesthetist and anaesthetic team administering the anaesthetic for ECT will not be blind, and will be aware of the randomisation by being able to identify the study drug at the time of ECT in the packaging provided by pharmacy. Once allocated, the patient will continue to receive the same experimental treatment during the study.

Baseline data

The information collected at baseline includes demographic and clinical data. The full list of baseline characteristics, including the neuropsychological tests and the efficacy ratings, is given in Section 2.2. Baseline neuropsychological and efficacy assessments will be set to missing if they are after the date/time of the first ECT. Missing baseline assessment data will not be imputed.

On-study assessments

Two ECT sessions are scheduled for each week and one to three days after every second ECT the subject should receive the efficacy rating assessments. After the fourth ECT (or if strictly necessary, after 3 or 5 ECT sessions), neuropsychological tests are performed. This is called the mid-course ECT neuropsychological assessment and constitutes the primary outcome time point.

While receiving acute ECT efficacy assessments will be carried out on a weekly basis. After the final acute ECT the efficacy and neuropsychological assessments will be performed +1day to +5 days after the final ECT, considered as the end ECT assessment.

Approximately four weeks (ranging from 3-5 weeks) after the end of acute ECT, the first follow-up visit will be performed, collecting efficacy and neuropsychological test data. Note, a subject could be on continuation ECT at this time. These assessments will then be repeated at 16 weeks (ranging from 12-20 weeks) after the end of acute ECT.

Neuropsychological outcome measures

Primary outcome measure:

- Hopkins Verbal Learning Test – Revised (HVLT-R) delayed recall (anterograde verbal memory, Trial 4)

Secondary outcome measures

- HVLT-R total learning (sum of correct responses for trials 1,2 and 3)
- HVLT-R retention [(Trial 4 ÷ higher score of trials 2 and 3)*100]
- HVLT-R recognition (total no. of true positives) - (total no. of false positives)
- Controlled Oral Word Association Test (COWAT) category fluency
- COWAT letter fluency
- Autobiographical Memory Interview – Short Form, modified scoring method, Semkovska et al (2012, AMI-SF SM2)
- AMI-SF, standard method of scoring, (AMI-SF SM1)
- Medical College of Georgia Complex Figure Test (MCGCFT) copy score
- MCGCFT immediate recall
- MCGCFT delayed recall
- Digit span Forward Correct repeats
- Digit span Backwards Correct repeats
- Global Self Evaluation of Memory (GSE-My) (Self-Reported)

Efficacy outcome measures

Main outcome measure

Montgomery-Åsberg Depression Rating Scale (MADRS) standard, i.e., total including 4a and 5a and omitting 4b and 5b (10 items)

Secondary outcome measures

- MADRS atypical, i.e., total including 4b and 5b (10 items as a and b versions are exclusive)
- Clinical Anxiety Scale (CAS)
  Total items 1-6; Total items 1-7 (which includes panic items)
- modified BPRS (including question 19 on elevated mood)
  Psychosis items from modified BPRS (sum of qs 3,4,7,8,11,12,15,16)
  Mania items from modified BPRS (sum of qs 8, 10, 17, 19)
- Remission (MADRS standard ≤10)
- Number of ECT treatments to achieve remission
- Response (≥ 50% decrease in standard MADRS from baseline)

- Clinical Global Impression – Severity (CGI-S)
- Clinical Global Impression - Improvement CGI-I)
- Quick Inventory of Depressive Symptomatology (QIDS-SR) (Self Report)
- From end ECT to follow up assessments: Proportion significantly worsening (MADRS increase of ≥4 points + CGI-S increase of ≥1 point to CGI-S ≥3 compared with end ECT assessment.
- EuroQol (EQ-5D)

Sample size and power calculations

Initial calculation: The study is designed to detect a standardised effect size (ES) of 0·53 between the ketamine treatment group and the placebo group in the primary outcome variable, HVLT delayed recall, after 4 ECT sessions. A sample size of 76 assessable patients per treatment group provides 90% power to detect this ES at a 5% significance level. Assuming 95% of patients can be assessed after 4 ECTs, this requires a total of 80 patients to be randomly assigned to each treatment group, or a total of 160. If only 85% of the 160 patients can be assessed then this gives 87% power to detect an ES of 0·53.

The three main cognitive interdependent measures are HVLT delayed recall, COWAT category fluency and AMI-SF. Based on a total of 76 assessable patients per group, and using a Bonferroni correction for the three outcomes, this gives 81% power to detect a standardised ES of 0·53 for all 3 outcomes assuming independence.

Revised power calculation September 2014: 90 patients (45 per treatment arm) gives 81% power to detect an ES of 0·6 for HVLT delayed recall. Depending on dropouts this will require between 90 (if 0% dropout) and 100 (if 10% dropout) patients to be recruited to achieve this at primary outcome.

Data description

Recruitment and representativeness of recruited patients

Consort chart to be added in here.

Baseline comparability of randomised groups

Patients in the two treatment groups will be described separately with respect to site, gender, age, ethnicity, marital status, occupation status, number of years in full-time education, highest academic qualification, family History of mental health, smoking and alcohol consumption. In addition, episode type, mood disorder type, co-morbid psychiatric disorders, degree of treatment resistance, age at onset of first mood episode or depression, number of prior depressive episodes, number of prior manic/hypomanic episodes, previous ECT therapy and inpatient / outpatient status will be summarised along with current physical co-morbidities (including whether due to cancer or congenital). Current psychiatric medication will be summarised. Handedness (mixed, left or right + score ratio), MMSE, and WTAR will also be summarised.

Numbers (with percentages) for binary, categorical variables and ordered categories will be presented. Means, standard deviations, and minimums and maximums for continuous variables will be presented.

Consistent with CONSORT guidance, there will be no tests of statistical significance or confidence intervals for differences between the randomised groups on any baseline variable.

All baseline neuropsychological and efficacy scales will be summarised assuming they are continuous variables (except for GSE-MY question 1 which is categorical), by treatment group. Summary statistics will be provided for each neuropsychological component captured on the CRF.

Treatment allocation questionnaire and treatment received

At the mid-course and end ECT treatment assessment the subject, ECT consultant/PI and RA are each asked which treatment they think the patient was allocated to and how certain they are about treatment allocation by choosing from one of four choices: pure guess, slight suspicion, moderately certain or very certain They also give the reason for their choice. The responses at the two time points will be tabulated by treatment arm.

A summary of how much treatment (total number of acute ECT sessions) received will be presented by treatment arm.

Treatment and trial discontinuation

The reasons for treatment discontinuation and study discontinuation / completion will be tabulated by treatment arm.

Assignment of neuropsychological assessments

As described in Section 1.6, the standard procedure is to perform two ECTs in the first week, a further 2 in the second week and then undertake the first neuropsychological assessment which is denoted as the mid-course ECT assessment which is the primary endpoint time. The Appendix illustrates rules for handling the ECT and neuropsychological data.

Neuropsychological and efficacy descriptives

The neuropsychological scales will be summarised for baseline, mid-course, end ECT and the two follow-up periods.

Efficacy scales will be summarised at baseline and then for each week while on acute treatment by arm. In addition the end ECT efficacy measure plus the two follow-up visits will be summarised.

Loss to follow-up

Selected baseline characteristics of subjects providing outcome measures after the mid-ECT session and those with missing data will be compared using a logistic regression model. Similarly, separate logistic regression models will be used to investigate patterns of failure to provide outcome measures after the final ECT and the two follow-up times, using both baseline characteristics and intermediate outcomes of treatment allocation (number of ECTs received and measures of both cognitive deficits and severity of depression). These analyses will be used to generate time-dependent inverse probability weights to evaluate the sensitivity of the formal analyses of outcomes to missing data (see below).

Formal analyses

The analyses comparing the ketamine and placebo arm will be conducted applying a modified intention to treat (ITT) approach. To be included in the modified ITT analyses a subject must have had at least 1 ECT (regardless of the quality).

If the degree of non-adherence to the ECT regime is substantial, and if failure to provide outcome data is associated with non-adherence, then the primary ITT analysis will be supplemented by estimation of the Complier-Average Causal Effect (CACE) of treatment using methods described in Dunn et al. (2005).

Differences in cognitive impairment

Cross-sectional analysis of covariance (ANCOVA) models (allowing for stratifying variables, age, sex, baseline degree of treatment resistance, electrode placement (bilateral or unilateral) and baseline values of the particular outcome being evaluated (if appropriate) will be used to evaluate the effects of treatment allocation on the neurocognitive test scores. If the subject withdraws from treatment or treatment ends after 3-5 sessions, the subsequent NP assessment will be assigned as “mid-ECT” with assignment to “end-ECT” dependent on reasons for discontinuing treatment as described in Appendix 1. If subjects are not able to be included due to lack of data inverse probability weighting adjustments will be used to assess the sensitivity of the findings to missing data (see above). All analyses will involve the use of robust standard errors and associated confidence intervals (allowing for non-normality and constraints in the ranges of some of the cognitive outcomes).

The main inference will be based on treatment effect for the HVLT cognitive assessment completed at the mid-course assessment. Statistical analysis of this outcome at the mid-course assessment will use a 5% two-sided significance level. Evaluation of treatment effects at the end of ECT and follow-up times will be regarded as secondary and the two-sided significance level for all other statistical tests will be 5%.

Differences in severity of depression

The MADRS weekly data will be analysed using a random effects (random intercepts and slopes) ANCOVA model with time (in weeks) from first ECT as a quantitative explanatory variable. The baseline variables will be the same as those for cognitive assessment. An interaction term between time and treatment allocation will also be included to assess the treatment effect. All analyses will use robust standard errors.

Note, if an end ECT efficacy measure is available then this will be assigned to a given week, yielding the last measure while on acute ECT used in the random effects analyses.

The CAS and QIDS-SR will be analysed using the same random effect modelling approach.

The binary outcomes will be analysed using longitudinal logistic regression.

Differences in number of ECT sessions provided

The number of ECT treatments to achieve remission will be analysed using a Poisson/negative binomial model for count data.

Exploratory analyses of end-ECT cognitive performance

If average cognitive impairment is less in the ketamine arm and also there have been fewer ECT sessions needed for remission in this arm this raises the question “Is impairment less in the ketamine arm because the participants have been exposed to fewer sessions of ECT (i.e. it is more effective), or is ketamine protective within each ECT session (or both)?” A simple pragmatic approach will be to stratify by the number of sessions received and to compare average cognitive performance across treatment arms within strata (testing whether there might be a dose-response effect). However here, we make the assumption that the ECT treatment has not been terminated (partly) because of the cognitive side-effects – which may not be justified – and even if it were, there is still the possibility that the effect of sessions on the difference between arms might be confounded.

ECT Treatment

All pre ECT data collected on the CRF will be summarised.

For each ECT session, means and standard deviations, plus minimums and maximums, will be presented by arm for continuous ECT treatment data i.e anaesthetic dose and units (separately for Propofol, Thiopental, Suxamethonium or other drug) and number of stimuli given. Electrode placement (bilateral vs unilateral) will be tabulated by stimulus number (1-4) by treatment group.

Post-ECT, the proportion of subjects getting 4 or more correct out of a total of 5 orientation questions at 30 and 60 minutes after first breath following ECT will be tabulated. In addition, tables showing the frequency of number correct (0-5) will be presented along with summary statistics for the number of correct items at 30 and at 60 minutes will be presented by arm.

Safety

Pre-ECT blood pressure and pulse will be summarised by treatment arm and also after each ECT treatment.

Adverse Events

To be handled by the research team in the Neuroscience and Psychiatry Unit

Statistical Analysis Plan References

Semkovska M, Noone M, Carton M, McLoughlin DC. Measuring consistency of autobiographical memory recall in depression. Psychiatry Res. 2012. 197:41-48.

Semkovska M, McLoughlin DM. Objective cognitive performance associated with electroconvulsive therapy for depression: a systematic review and meta-analysis. Biol Psychiatry. 2010 68:568-77.

Dunn G, Maracy M, Tomenson B. Estimating treatment effects from randomized clinical trials with noncompliance and loss to follow-up: the role of instrumental variable methods. Statistical Methods in Medical Research. 2005. 14:369-395.

Appendix to statistical analysis plan: Programmatic rules for handling ECT and neuropsychological (NP) outcome data

Note these rules are for the analysis and differ to the procedural specification.

a. Ideally the baseline NP assessment should be ≤ the randomisation date but must be before the subjects first ECT. The assessment standard operating procedure E1 (SOPE1) states that the baseline NP assessment should be -2weeks to -1day preECT1. For analysis all baseline assessments will be included unless there is an indication that the clinical condition has markedly changed before ECT1 (in practice this has been assessed prospectively and if the clinical condition has changed the RAs re-do the baseline).
b. The first, and priority, step is to establish whether there is a valid primary outcome ("mid-ECT") NP assessment during the acute course of ECT. To do this the number of acute course ECTs prior to the first post-baseline NP assessment will be calculated. If the subject has had 3-5 prior ECTs then this NP assessment is assigned as the "mid-ECT" assessment, regardless of missed ECT sessions. SOPE1 states this NP assessment should be between 1 and 3 days after the prior ECT session. For analysis, if the assessment is >5 days then this will be treated as missing (based on cognitive effects of ECT likely to have substantially resolved). A sensitivity analysis will be carried out omitting subjects with assessments at 4 or 5 days (based on Semkovska & McCloughlin 2010).
c. The "end-ECT" NP assessment is assigned if the next NP assessment is completed after the end of the ECT acute treatment course and should be between 1 and 5 days after the last ECT treatment date according to SOPE1. For analysis, if the assessment is >12 days this will be treated as missing (based on cognitive effects of ECT likely to have resolved). NB this is longer than for the primary outcome based on the uncertainty as to when cognitive effects from ECT resolve, however a sensitivity analysis will be carried out omitting subjects with assessment at 6-12 days (based on Semkovska & McCloughlin 2010).
d. In the unlikely event that acute ECT finishes after 1 or 2 treatments the NP assessment will be assigned as "mid-ECT". In addition, a sensitivity analysis will be performed by omitting these subjects and estimating the treatment effect.
e. If the subject withdraws from treatment or treatment ends after 3-5 sessions, the subsequent NP assessment will be assigned as both "mid-ECT" and "end-ECT" when ECT has been stopped due to sufficient clinical response, and as "mid-ECT" only if treatment has stopped for other reasons ("end-ECT" will be treated as missing data in this case).
f. The Follow up 1 month after end of ECT acute treatment course (FU1) should be between 3 and 5 weeks after the last ECT of the acute treatment course, and at least 2 weeks after stopping any continuation ECT treatment, according to SOPE1. NB the latter can't be checked programmatically as continuation ECT is not recorded on the database but is recorded by the project manager. For analysis if there is no FU1 then any end ECT assessment >14 days and <6 weeks after last ECT will be assigned as FU1 if there is no FU1 recorded. In the unlikely case that FU1 is also available the assessment closest to 1 month after last ECT will be included and the other will be excluded.
g. SOPE1 states that follow up 4 months after end of ECT acute treatment course (FU2) should be between 12 and 20 weeks after the last ECT of the acute treatment course. For analysis any assessment at least 8 weeks after the last ECT will be included.

Supplementary results

Sensitivity analyses

HVLT-R-DR sensitivity analysis excluding assessments four and five days after the last ECT at Mid-ECT (n=4) and assessments 6-12 days after the last ECT at End of Treatment (n=11):

Mid-ECT	-0·56 (95%CI -1·91 to 0·79)
End of Treatment	-0·56 (95%CI -1·82 to 0·71)
One month follow-up	-0·53 (95%CI -1·65 to 0·60)
Four month follow-up	-1·43 (95%CI -2·95 to 0·10)

MADRS sensitivity analysis based on time since randomisation instead of time since first ECT:
Difference in treatment slopes -0·54 (95% CI -1·94 to 0·85), p=0·45

Note: negative values favour saline

**Measure**	**Ketamine**		**Saline**	
		**n**		**n**
**AMI-SF percentage of baseline**				
Mid-ECT	85·5 (10·8)	29	86·3 (12·4)	36
End of Treatment	76·3 (15·3)	25	77·4 (16·1)	32
1 month follow-up	77·8 (14·9)	23	76·9 (14·7)	23
4 month follow-up	82·4 (10·2)	19	83·3 (14·4)	16

Values are means (SD) and n for valid assessments at each time point. AMI-SF=Autobiographical Memory Interview-Short Form

***Supplementary Table 1*: AMI-SF as percentage of baseline in patients receiving ECT randomised to ketamine or saline**

Measure	Ketamine		Saline		Repeated measures analysis[a]	
	**Mean (SD)**	**n**	**Mean (SD)**	**n**	**Treatment effect difference (95% CI)**	**p**
**Present memory**						
Baseline	3·73 (1·15)	33	3·57 (1·32)	37		
Mid-ECT	3·61 (1·23)	28	3·58 (1·13)	36	0·05 (-0·49 to 0·58)	0·86
End of Treatment	3·27 (1·19)	26	3·63 (1·13)	32	-0·29 (-0·85 to 0·28)	0·32
Follow-up 1	3·87 (1·22)	23	4·13 (1·42)	23	-0·28 (-0·99 to 0·44)	0·45
Follow-up 2	3·53 (1·26)	19	4·39 (1·50)	18	-0·68 (-1·33 to 0·27)	0·092
**Effect of ECT[b]**						
Baseline (expectation)	3·73 (1·15)	33	3·61 (1·38)	37		
Mid-ECT	3·64 (1·29)	29	3·17 (1·07)	36	0·13 (-0·35 to 0·61)	0·60
End of Treatment	3·36 (0·87)	26	2·94 (1·08)	32	0·27 (-0·35 to 0·89)	0·40
Follow-up 1	3·12 (1·24)	23	3·09 (1·11)	23	-0·01 (-0·70 to 0·43)	0·97
Follow-up 2	3·09 (1·04)	19	3·53 (1·12)	18	-0·53 (-1·33 to 0·27)	0·20
**Has ECT affected your memory?**	**n (%)**	**n**	**n (%)**	**n**		
Mid-ECT						
Yes better	1 (3%)		3 (8%)			
No	13 (45%)	29	10 (28%)	36		
Yes worse	15 (52%)		23 (64%)			
End of Treatment						
Yes better	3 (12%)		2 (6%)			
No	7 (27%)	26	7 (22%)	32		
Yes worse	16 (62%)		23 (72%)			
Follow-up 1						
Yes better	2 (9%)		2 (9%)			
No	6 (25%)	23	5 (22%)	23		
Yes worse	15 (65%)		16 (70%)			
Follow-up 2						
Yes better	1 (5%)		2 (11%)			
No	7 (37%)	19	9 (50%)	18		
Yes worse	11 (58%)		7 (39%)			

Means, %s and ns refer to observed valid assessments at each time point. Ratings on a 1-7 scale with 4 so-so (present memory) or no-change (effect of ECT on memory); >4 better and <4 worse.

[a] mITT analysis adjusting for age at randomisation, sex, baseline degree of treatment resistance, electrode placement (bilateral or unilateral) and baseline value. Negative values favour saline.

[b] Baseline value is expectation of the effect of ECT, at other time points are how much ECT has affected memory.

***Supplementary Table 2*: Global self-evaluation of memory (GSE-My) in patients receiving ECT randomised to ketamine or saline**

	Ketamine n=33			Saline n=37		
		Cumulative			Cumulative	
Week	n	n	(%)	n	n	(%)
1	0	0	0.0	1	1	2.7
2	2	2	6.1	5	6	16.2
3	1	3	9.1	0	6	16.2
4	2	5	15.2	3	9	24.3
5	2	7	21.2	1	10	27.0
6	3	10	30.3	3	13	35.1
7	1	11	33.3	0	13	35.1
8	1	12	36.4	0	13	35.1
9	0	12	36.4	0	13	35.1
10	1	13	39.4	0	13	35.1

Supplementary Table 3: **Number and cumulative frequency of remissions while receiving ECT therapy**

	Ketamine n=33			Saline n=37		
		Cumulative			Cumulative	
Week	n	n	(%)	n	n	(%)
1	1	1	(3.0)	2	2	(5.4)
2	2	3	(9.1)	5	7	(18.9)
3	5	8	(24.2)	6	13	(35.1)
4	2	10	(30.3)	2	15	(40.5)
5	3	13	(39.4)	4	19	(51.4)
6	0	13	(39.4)	3	22	(59.5)
7	2	15	(45.5)	0	22	(59.5)
8	0	15	(45.5)	0	22	(59.5)
9	1	16	(48.5)	0	22	(59.5)

Supplementary Table 4: **Number and cumulative frequency of responders while receiving ECT therapy**

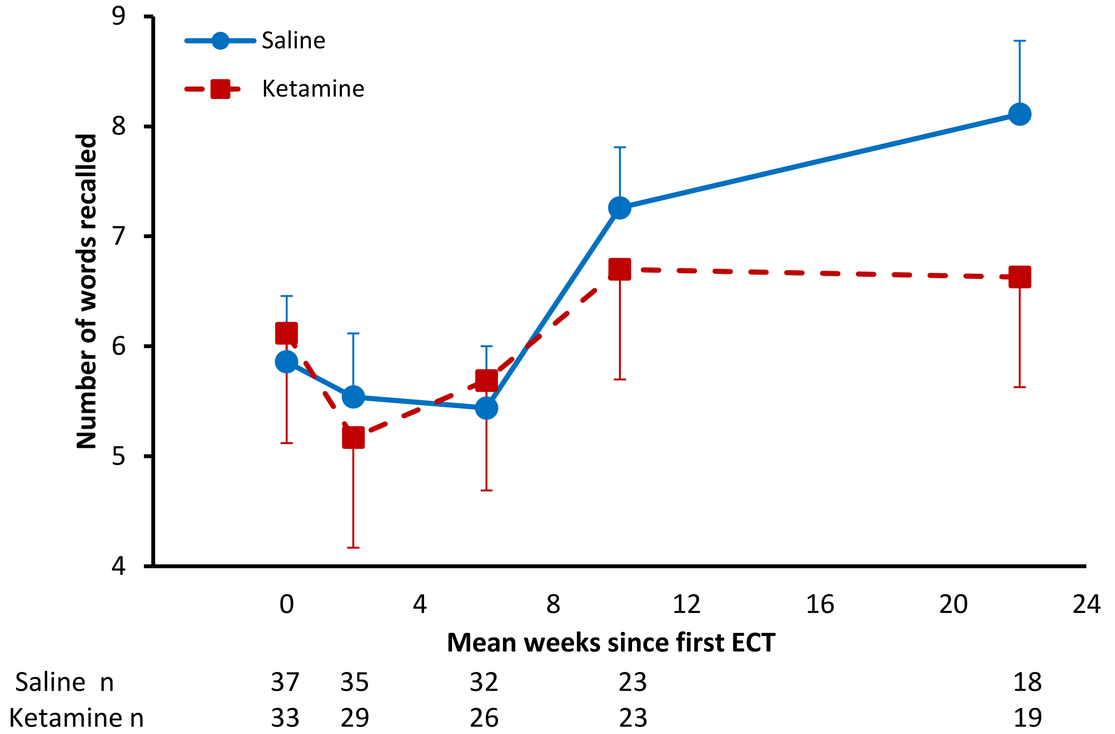

Supplementary Figure 1: **Mean HVLT delayed recall score during ECT and follow-up**

Data points are mean time from first ECT for Mid-ECT, End of Treatment and Follow up assessments. Values under the x-axis show number of participants with valid assessments at each time point. Error bars show SEM

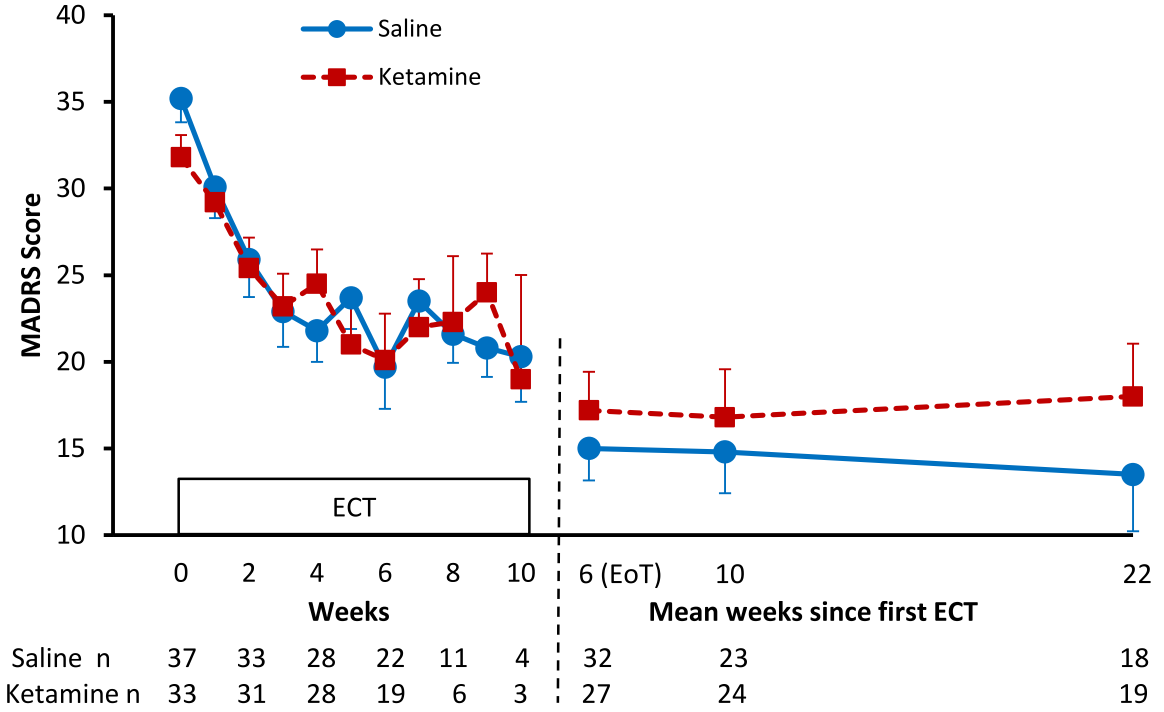

Supplementary Figure 2: **Mean Montgomery Åsberg Depression Rating Scale scores during ECT and follow up.**

EoT=End of Treatment. Data points on left half of Figure are assessment weeks during ECT treatment excluding one participant on saline who continued ECT to 13 weeks. Data points on the right of Figure are mean time from first ECT for End of Treatment and Follow up assessments. Values under the x-axis show number of participants with valid assessments at each time point. Error bars show SEM

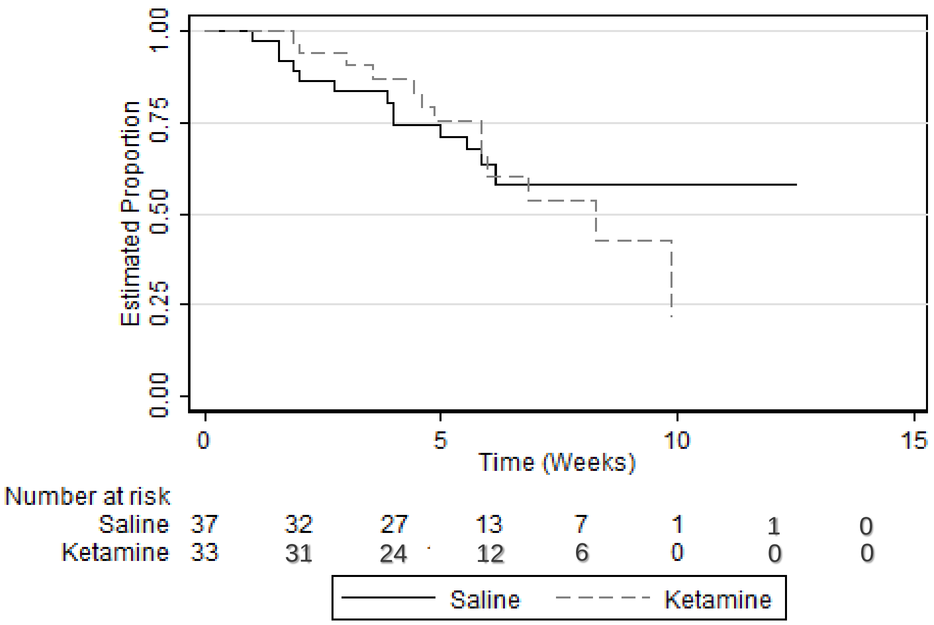

Supplementary Figure 3: **Kaplan Meier plot of time from first ECT to first remission by group.** Remission defined as MADRS ≤10. Hazard ratio 1.16 (95% CI 0.51 to 2.64) p= 0.73.

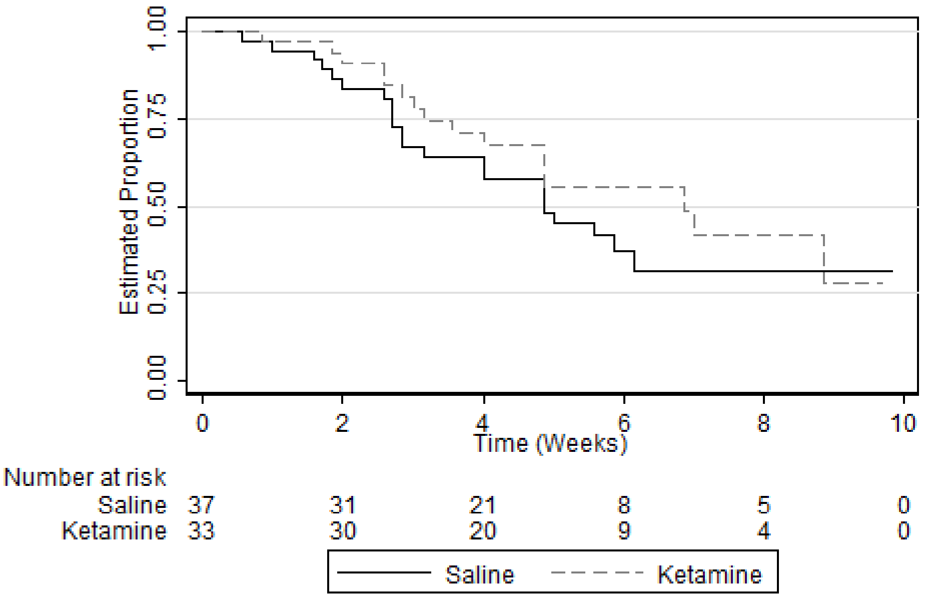

Supplementary Figure 4: **Kaplan Meier plot of time from first ECT to first response by group.** Response defined as decrease in MADRS by ≥50%. Hazard ratio 0.99 (95% CI 0.49 to 1.99) p=0.97.
